# Editorial: Guardians of protein homeostasis (proteostasis) in health, disease and aging

**DOI:** 10.3389/fmolb.2023.1350666

**Published:** 2023-12-19

**Authors:** David A. Dougan, Kaye N. Truscott, Janine Kirstein

**Affiliations:** ^1^ Department of Biochemistry and Chemistry, La Trobe Institute for Molecular Science, La Trobe University, Melbourne, VIC, Australia; ^2^ Faculty of Biosciences, Institute of Biochemistry and Biophysics, Friedrich-Schiller-Universität Jena, Jena, Germany; ^3^ Leibniz-Institute on Aging/Fritz-Lipmann Institute, Jena, Germany

**Keywords:** Hsp70, AAA+ proteins, JDP, ATP-dependent unfolding, proteostasis, protein remodelling, chaperone, degradation

## Introduction

Protein homeostasis, also known as proteostasis, refers to the dynamic balance of functional proteins within the cell, required to support normal cellular activities. It is a type of equilibrium, that encompasses every facet of a protein’s lifetime, commencing with its synthesis on the ribosome and subsequent folding, to modifications, transport and assembly formation and culminating in its breakdown through degradation. Proteostasis is a finely regulated processes that is actively maintained by an extensive network of molecular components (including molecular chaperones and proteases) collectively known as the proteostasis network (Balchin et al., 2016). The network plays a central role in maintaining the overall health and function of a cell, not only under normal conditions, but also under conditions that cause protein damage otherwise known as proteotoxic stress. The maintenance of the cellular protein population in its native folded and functional state is central to proteostasis, with the loss of proteostasis marked by the accumulation of dysfunctional misfolded and aggregated proteins.

At its essence, the proteostasis network manages two fundamental processes; one that controls the synthesis and conformational maintenance of the proteome, ensuring the proper folding and structural integrity of a protein, and the other that is responsible for the removal of irreversibly misfolded, damaged or unwanted proteins through proteolysis. This dynamic equilibrium ensures that the functional, folded protein state predominates. This Research Topic, focuses on the role of two prominent families within the proteostasis network i) the Hsp70 (Heat shock protein 70) chaperone family that, together with its co-chaperones, protects the cell from protein conformational diseases (Rosenzweig et al., 2019) and ii) the AAA+ (ATPase associated with a variety of cellular activities) superfamily of proteins (Neuwald et al., 1999) that are largely, but not exclusively, associated with the unfolding of damaged or unwanted proteins in preparation for their removal through degradation. This Research Topic, comprised of four reviews and one original research article, provides a comprehensive understanding of the strategies used by these distinct protein families to protect the cell from the toxic effects of proteostasis collapse.

### Cellular protein homeostasis is supported by balanced activities of Hsp70 and the ubiquitin proteasome system

The proteostasis network encompasses three main strategies to combat proteotoxic stress associated with misfolded proteins; i) refolding and disaggregation, ii) degradation and iii) sequestration. In an original study, using the model yeast, *Saccharomyces cerevisiae*, Mogk and colleagues (Jawed et al.) explored the intricate relationship between these different strategies. Specifically, they generated mutant yeast cells lacking two Hsp70 partner chaperones and the nuclear sequestrase (Btn2) and examined the role of the Ubiquitin Proteasome System (UPS) in these cells. Remarkably, these mutant yeast cells (with reduced Hsp70 capacity) exhibited a breakdown of proteostasis due to a strong imbalance between refolding and proteolytic activities, resulting from a global activation of various stress responses and induction of the UPS. Significantly, reducing the activity of the UPS in these Hsp70 capacity mutants, mitigated stress responses and restored protein homeostasis, demonstrating the importance of precise regulation of the proteostasis network and the significance of an equilibrium amongst the different proteostasis modules (Balch et al., 2008). Intriguingly, although proteostasis balance appeared to be restored in these cells through changes in the levels of Hsf1, the mechanism by which this occurs is currently unclear. Hence further research to elucidate the mechanism of Hsf1 upregulation is eagerly awaited.

### Targeting of client proteins to the ATP-dependent unfoldase, p97

p97 (also known as VCP or Cdc48) is a highly abundant AAA + protein that serves as a critical molecular hub for the unfolding of hundreds of structurally and functionally diverse client proteins in eukaryotic cells (Meyer et al., 2012; Buchberger et al., 2015). Given the broad variety of its client proteins (and the cellular pathways they are involved in), it is unsurprising that p97 is an important component of the proteostasis network. To maintain the functional diversity of this machine, while retaining its client protein specificity, p97 activity is mediated by several co-factors and adapters. In this Research Topic Meyer and van der Boom provide a comprehensive overview of the primary mechanisms used by p97 to achieve these diverse cellular tasks. In general, p97 employs two alternative mechanisms for the targeting of client proteins, each of which is facilitated by mutually exclusive client adapter proteins. The first mechanism involves the targeting of ubiquitylated client proteins to the UPS for their turnover by the 26S proteasome. This mechanism is facilitated by the heterodimeric adapter, Ufd1-Npl4 which, in some cases, also cooperates with additional accessory adapters such a FAF1 and UBXN7. The second mechanism concerns the remodelling of protein complexes through the dissociation of client proteins from a binding partner. This pathway involves the direct targeting of client proteins, by SEP (shp1, eyc and p47)-domain adapters such as p37, in a ubiquitin-independent manner.

### Reversible protein assemblies in the proteostasis network

Proteins exist in a broad range of conformations, from well-folded structures to entirely disordered regions. The conformational complexity of the proteome is further increased by the presence of higher-oligomeric protein assemblies, formed through stochastic and regulated interactions. Some of these protein assemblies can occur through liquid-liquid phase separation (LLPS) and are involved in an increasing number of physiological pathways (Wang et al., 2021). Conversely, protein aggregates have been conventionally seen as disordered structures formed spontaneously by misfolded proteins and are often associated with cellular dysfunction and stress. Recent insights have revealed that protein condensates formed through LLPS can mature into solid structures that harbour misfolded proteins and are decorated with proteostasis network factors. In this review, Kohler and Andreasson provide an informed comparison of biomolecular condensates and protein aggregates, highlighting the relationship between these two protein assemblies. While biomolecular condensates are crucial for cellular function, aging can transform them into solid-like structures with misfolded proteins, blurring the distinction between condensates and aggregates. The review discusses the cellular strategies that regulate these processes, emphasizing the pivotal role of disaggregation in determining the fate of proteins between refolding and degradation pathways.

### Alternative ATPase domain interactions in eukaryotic Hsp70

The Hsp70 family of molecular chaperones plays a crucial role in maintaining cellular protein balance. They generally interact with client proteins through their substrate binding domain (SBD) in a process that is regulated by the binding and hydrolysis of ATP (at the nucleotide binding domain (NBD)) and assisted by co-chaperones (Kampinga and Craig, 2010; Mayer and Gierasch, 2019). However, recent research has identified a handful of proteins that interact with select Hsp70s in a way that can neither be classified as co-chaperones nor clients as they do not fit to the classical ATP- regulated client interaction. In this review, Khoud et al. termed this class of interacting proteins as Hsp70 ATPase alternative binding (HAAB) proteins. Although the molecular details of these interactions are yet to be fully understood, the review highlights the emerging features of these proteins and their interaction with Hsp70s. The study underscores the versatile functions of Hsp70 chaperones and expands on recent discoveries demonstrating that Hsp70 interacts with numerous proteins throughout its domains and that those interactions are regulated by post-translational modifications (Nitika et al., 2022).

### Pathological exploitation of proteostasis machinery by exported J-domain proteins

Proteostasis relies on a network of molecular chaperones and co-chaperones. The J domain protein (JDP) family is a diverse group of co-chaperones that plays a crucial role in modulating the activity of Hsp70 chaperones (Kampinga and Craig, 2010; Zhang et al., 2023). This collaboration forms a potent protein quality control network within cells. In the context of *P. falciparum* (the malaria parasite responsible for invading and transforming human erythrocytes) there is a notable involvement of both human and parasite chaperone machineries. Malaria-infected erythrocytes exhibit high levels of functional human Hsp70, with lower levels of human JDPs. Intriguingly, *Plasmodium falciparum* JDPs (PfJDPs) are the most prominent and diverse family of proteins that are exported into the infected erythrocyte cytosol, suggesting they may play a crucial role in the parasite’s survival and pathogenesis. In this review, Blatch and colleagues critically evaluate the current understanding of how exported PfJDPs exploit the proteostasis machinery, fine-tuning the chaperone properties of both human and malarial Hsp70s in the context of malaria pathogenesis. They discuss evidence that points to a complex network of interactions between PfJDPs, the exported malarial Hsp70 (PfHsp70-x), and human Hsp70, which is implicated in the trafficking of key malarial virulence factors and the maintenance of proteostasis for protein complexes associated with pathology.

## Concluding remarks

In conclusion, this focussed Research Topic provides a general overview of select members of the proteostasis network illustrating, not only, the physiological importance of these systems in protecting the cell from protein conformational disease but also exploring the role of these systems in the context of host-pathogen interactions. The Research Topic highlights some of the recent developments employed by these proteostasis network members to maintain cellular health, from emerging and non-classical roles of these systems to mechanistic details describing how these systems function both individually and in cooperation with one another.
